# The role of the muscarinic system in regulating estradiol secretion varies during the estrous cycle: the hemiovariectomized rat model

**DOI:** 10.1186/1477-7827-4-43

**Published:** 2006-08-21

**Authors:** María E Cruz, Angélica Flores, María T Palafox, Griselda Meléndez, Jorge O Rodríguez, Roberto Chavira, Roberto Domínguez

**Affiliations:** 1 Unidad de Investigación en Biología de la Reproducción. FES Zaragoza. UNAM, México; 2 Instituto Nacional de Ciencias Médicas y de la Nutrición "Salvador Zubirán," México

## Abstract

There is evidence that one gonad has functional predominance. The present study analyzed the acute effects of unilateral ovariectomy (ULO) and blocking the cholinergic system, by injecting atropine sulfate (ATR), on estradiol (E_2_) serum concentrations during the estrous cycle. The results indicate that ULO effects on E_2 _concentrations are asymmetric, vary during the estrous cycle, and partially depend on the cholinergic innervation.

Perforation of the left peritoneum resulted in lower E_2 _serum concentrations in the three stages of the estrous cycle. At proestrus, unilateral or bilateral perforation of the peritoneum resulted in lower E_2 _serum concentrations.

ULO of the right ovary (left ovary *in situ*) resulted in significantly higher E_2 _concentrations than animals with ULO of the left ovary (right ovary *in situ*). ATR treatment to ULO rats on D1 resulted in a significant drop of E_2 _serum concentrations. ULO rats treated with ATR on D2 or P, resulted in an asymmetrical E_2_ secretion response; when the right ovary remained in situ an increase in E_2_ was observed, and a decrease when the left ovary remained *in situ*.

The results obtained in the present study suggest that each ovary's ability to compensate the secretion of E_2 _from the missing ovary is different and varies during the estrous cycle. The results also suggest that the cholinergic system participates in regulating ovarian E_2 _secretion. Such participation varies according to the ovary remaining *in situ *and the stage of the estrous cycle of the animal.

The results agree with previously stated hypothesis of a neural pathway arising from the peritoneum that participates in regulating E_2 _secretion, and also supports the idea of cross-talk between the ovaries, via a neural communication, that modulates E_2 _secretion.

## Background

Estradiol secretion is regulated by pituitary [follicle stimulating hormone (FSH) and luteinizing hormones (LH), prolactin, and adrenocorticotropin (ACTH)]. The effects of these hormones are modulated by neurotransmitters released by the intrinsic ovarian innervation near the follicular wall. Acetylcholine produced by the follicle may be one of the neurotransmitters participating in modulating the effects of pituitary hormones on the follicle [1-3].

Evidence suggesting that one gonad has functional predominance in mammals and birds have been published [1,4-8]. In previous studies we have shown that unilateral ovariectomy (ULO) modifies progesterone and/or testosterone serum concentrations, and that the effects of ULO depend on both, the stage of the estrous cycle when ULO was performed and the ovary (left or right) remaining *in situ *[9-11].

Asymmetry in ovarian functions has been explained by differences in the ovarian innervation participating in modulating the effects of gonadotropin on the ovarian follicles [1,6]. Kawakami et al [12] showed that electrical stimulation of the medial basal pre-chiasmatic area, the ventro-medial hypothalamus, and the areas in the mesencephalon of hypophysectomized and adrenalectomized female rats resulted in a significant increase of estradiol (E_2_) and progesterone (P_4_) plasma concentrations in the contra-lateral ovarian venous blood. In turn, stimulating the dorsal hippocampus, the lateral amygdala, and the mesencephalic areas resulted in lower E_2 _and P_4 _concentrations. Ovarian denervation of rats in proestrus stage blocks E_2 _secretion induced by stimulating the medial basal pre-chiasmatic area. In addition, the electrochemical stimulation in proestrus day of the medial basal pre-chiasmatic area of untreated rats increased E_2 _and P_4 _concentrations in serum. This effect was not observed when stimulation was applied to the pre-optic supra-chiasmatic area. According to the authors' interpretation of the results, the efferent neural system connecting the brain and the ovaries is supplementary to the brain-pituitary-ovarian hormonal mechanisms regulating ovarian steroid secretion, and the system may be required for adjusting ovarian responsiveness and sensitivity to gonadotropins [12,13].

Gerendai et al. [14] described a multi-synaptic neural pathway between the central nervous system and the ovaries, with the vagus nerve being one of the main neural pathways. In ULO treated rats, bi-lateral sectioning the vagus nerve (ventral or dorsal) results in lower compensatory ovarian hypertrophy. The effects of sectioning the left vagus nerve depend on the remaining ovary *in situ*: rats with the left ovary *in situ *had a larger proportion of ovulating animals, compensatory ovarian hypertrophy and number of ova shed. In turn, rats with the right ovary *in situ *showed a decrease in all parameters studied [15].

Based on available information, the present study aims to analyze if changes in E_2 _secretion by the left and right ovaries vary during the estrous cycle, using the unilateral ovariectomized animal as a model of study.

We also investigated if, throughout estrous cycle diestrus 1 (D1), diestrus 2 (D2) and proestrus (P), the cholinergic system modulates E_2 _secretion in an asymmetric way. For this purpose, we analyzed the effects of injecting ATR at 13.00 h to rats on D1, D2 or P with or without unilateral or bilateral ovariectomy.

## Materials and methods

The study was performed with virgin adult female rats (195–225-g body weight) of the CIIZ-V strain from our own stock. Animals were kept under controlled lighting conditions (lights on from 05:00 to 19:00 h), with free access to food (Purina S.A., Mexico) and tap water; following NIH Guide parameters for the care of laboratory animals. The Committee of the Facultad de Estudios Superiores Zaragoza approved the experimental protocols.

Estrous cycles were monitored by daily vaginal smears. Only rats showing at least two consecutive 4-day cycles were used in the experiment. All surgeries were performed under ether anesthesia, between 13:00–13:15 hours. Rats were sacrificed by decapitation one hour after treatment.

### Experimental groups

Rats were randomly allotted to one of the experimental groups described below. Animals from different experimental groups were treated simultaneously and sacrificed one hour after surgery. The number of animals used in each experimental group is presented in Tables 1, 2 and 3.

**Table 1:** Effects of left, right or bilateral peritoneum perforation (PP) performed at 13:00 h of diestrus 1, diestrus 2 or proestrus on estradiol serum levels (pg/ml) measured one hour after surgery. Data are represented by means ± SEM.

**Group**	**N**	**Diestrus 1**	**N**	**Diestrus 2**	**N**	**Proestrus**
**Control**	18	55.3 ± 8.0	20	59.1 ± 7.9	15	158.4 ± 10.8
**Left PP**	8	25.0 ± 4.5*	10	29.1 ± 8.2*	9	111.1 ± 15.3*
**Right PP**	11	46.7 ± 7.5	9	109.3 ± 5.5*	10	78.9 ± 8.3*
**Bilat. PP**	8	76.3 ± 6.5	10	63.3 ± 7.1	9	93.7 ± 16.8*

* p < 0.05 vs. control group (ANOVA followed by Tukey's test)

**Table 2:** Means ± SEM of estradiol serum concentration (pg/ml) in animals with bilateral perforation of the peritoneum (PP) or bilateral ovariectomy (CAS) performed at 13:00 h on Diestrus 1, Diestrus 2 or Proestrus, sacrificed one hour later

**Group**	N	**Diestrus 1**	N	**Diestrus 2**	N	**Proestrus**
**Bilateral PP**	8	76.3 ± 6.5	10	63.3 ± 7.1	9	93.7 ± 16.8
**CAS**	7	20.2 ± 3.7*	10	17.3 ± 2.6*	6	14.7 ± 5.4*

* p < 0.05 vs. bilateral PP group (Student's t test)

**Table 3:** Means ± SEM. of estradiol serum concentration (pg/ml) in control and animals injected with atropine sulfate (ATR) at 12:00 h on Diestrus 1, Diestrus 2 or Proestrus. The animals were sacrificed at 14:00 h.

***Group***	N	**Diestrus 1**	N	**Diestrus 2**	N	**Proestrus**
**Control**	17	55.3 ± 8.0	20	61.7 ± 4.9	12	158.4 ± 10.8
**ATR**	8	9.0 ± 1.8*	8	67.5 ± 2.7	7	51.7 ± 9.8*

* p < 0.05 vs. non-ATR treated group (Student's t test)

Control group (*N *= 48). Non-treated cyclic rats sacrificed at 14:00 h on D1 (17 rats), D2 (19 rats) and P (12 rats).

Ether anesthesia (*N *= 24): Groups of rats, on specific stages (D1, D2 or P) of the estrous cycle, were anesthetized for 10 min and sacrificed one hour later.

Unilateral peritoneal perforation (sham operation) (*N *= 53): A unilateral incision was performed 2-cm below the last rib; affecting skin, muscle, and peritoneum. The ovaries were not injured or manipulated. After surgical procedures the wound was sealed.

Bilateral peritoneal perforation (sham operation 2) (N = 27). A bilateral incision below the last rib, including skin and muscle, was performed. The ovaries were not injured or manipulated. After surgical procedures the wound was sealed.

Unilateral ovariectomy (ULO) (N = 50): A unilateral incision below the last rib, including skin and muscle was performed, and the right or left ovary was extirpated. The wound was subsequently sealed.

Bilateral ovariectomy (*N *= 23): A bilateral incision below the last rib, including skin and muscle was performed, and the ovaries removed. The wound was subsequently sealed.

### Blocking the cholinergic system

To analyze the effects of blocking the cholinergic system, groups of animals were injected with atropine sulfate (ATR, Sigma Chem. Co. St. Louis, Mo.). ATR was injected one hour before surgery at doses known to block ovulation: in D1, 100 mg/kg body weight (b.w.); in D2, 300 mg/kg b.w.; and in P, 700 mg/kg b.w. [16].

One hour after ATR treatment, rats were randomly allotted to one of the following treatments: unilateral peritoneal perforation, bilateral peritoneal perforation, ULO, or bilateral ovariectomy. All animals were sacrificed one hour after surgery. For control purposes, untreated rats, on D1, D2 or P, were injected with ATR in the same dose as in their corresponding treatment group. The animals were sacrificed two hours after treatment.

### Autopsy procedures

Animals were sacrificed by decapitation. The blood of the trunk was collected in a test tube, allowed to clot at room temperature for 30 minutes and centrifuged at 3,000 rpm for 15 minutes. Serum was stored at -20°C, until E_2_ concentrations were measured.

### Hormone assay

Concentrations of E_2 _in serum were measured by Radio-Immuno-Assay (RIA); using kits purchased from Diagnostic Products (Los Angeles, CA). Results are expressed in pg/ml. The Intra- and inter-assay variation coefficients were 6.9% and 10.8 %, respectively.

### Statistics

Data on hormonal concentrations in serum were analyzed using multivariate analysis of variance (MANOVA) followed by Tukey's test. Differences in serum hormone concentrations between two groups were analyzed by Student's *t*-test. A probability value of less than 5% was considered significant.

## Results

### Effects of ether anesthesia and unilateral or bilateral perforation of the peritoneum

In the control group, animals sacrificed on P showed significantly higher E_2 _serum concentration than animals sacrificed on D1 or D2 (D1: 55.3 ± 8.0; D2: 59.1 ± 7.9; P: 158.4 ± 1.8). Compared to the control group, ether anesthesia treatment did not modify E_2 _serum concentrations (D1: 62.9 ± 8.4; D2: 69.5 ± 12.0; P: 164.1 ± 17.6). Since ether anesthesia did not modify E_2 _serum concentrations, treatment results are compared to their respective control group.

The effects on E_2 _serum concentrations of unilaterally or bilaterally perforating the peritoneum depended on the side of the peritoneum and the stage of the estrous cycle when perforation surgery was performed. Perforating the left peritoneum on D1 resulted in lower E_2 _serum concentrations (55%), while bilateral perforation, or perforating the right side of the peritoneum, had no apparent effects (Table 1).

Perforating the right side of the peritoneum on D2 day resulted in E_2 _concentration increases (184%), while perforating the left side resulted in a decrease (51%) of E_2 _serum concentrations. Bilateral perforation had no apparent effects on hormone concentrations. Perforating the peritoneum on P phase (left, right or bilateral) resulted in hormone serum concentration decreases (Left 30%; Right 50%; Bilateral 41%). Results are summarized in Table 1.

### Effects of unilateral or bilateral ovariectomy

**Figure 1:**
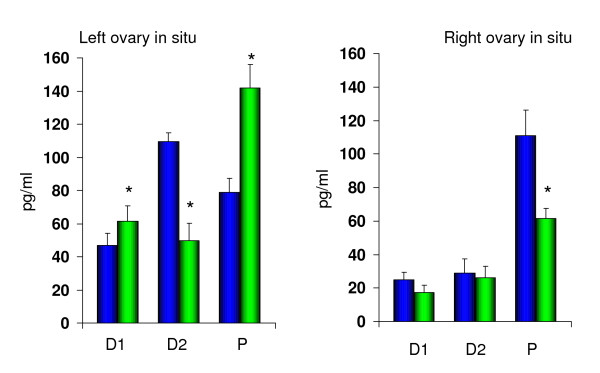
Effects of unilateral perforation of the peritoneum (PP) (blue) and unilateral ovariectomy (ULO) (green) on estradiol serum levels (pg/ml) performed at 13:00 h of diestrus 1 (D1), diestrus 2 (D2) or proestrus (P), sacrificed one hour later. * p < 0.05 vs. PP (Student's *t *test).

When surgery was performed on D1, no significant differences in E_2 _serum concentrations were observed between rats with ULO (animals with intact left or right ovary *in situ*) or perforation of the peritoneum (Figure 1). Animals with the left intact ovary *in situ *showed significantly higher E_2 _serum concentrations than animals with the right intact ovary *in situ *(61.5 ± 9.4 vs. 17.3 ± 4.3, p < 0.05 Student's t test).

Compared to animals with unilateral perforation of the peritoneum, animals with right ULO (left ovary *in situ*) performed on D2 had lower E_2 _serum concentrations (55%). Such differences were not observed in rats with left ULO (Figure 1). As in rats treated on D1, E_2 _serum concentrations were significantly higher in animals treated on D2 with the left ovary *in situ *(right ULO) than in animals with the right ovary *in situ *(49.5 ± 10.8 vs. 26.0 ± 6.9).

In animals treated on P, right ULO (left ovary *in situ*) resulted in higher E_2 _(180%) serum concentrations than in animals with unilateral peritoneum perforation. ULO performed on the left side (right ovary *in situ*), resulted in significantly lower (45%) E_2 _serum concentrations compared to rats with a unilateral perforation of the peritoneum (Figure 1). As observed in rats treated on D1 or D2, when the intact left ovary remains *in situ*, estradiol serum concentrations were significantly higher than in animals with the intact right ovary *in situ *(142.0 ± 14.1 vs. 61.5 ± 6.0). Compared to animals with a bilateral perforation of the peritoneum, bilateral ovariectomy resulted in significantly lower E_2 _serum concentrations, regardless of the stage of the estrous cycle surgery performed (D1 74%; D2 73%; P 84%). Results are summarized in Table 2.

### Effects of blocking the cholinergic system

Injecting ATR on D1 or P resulted in E_2 _serum concentrations decreases (84% and 67%, respectively), and had no apparent effects on E_2 _serum concentrations when injected on D2 (Table 3).

**Figure 2:**
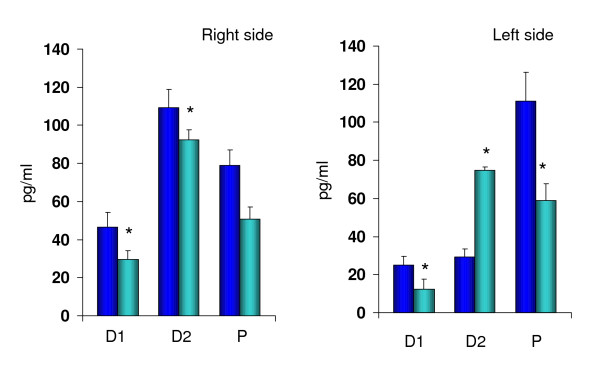
Comparative effects of atropine sulfate injection (blue) at 12:00 hours on estradiol serum levels (pg/ml), to rats with unilateral perforation of the peritoneum (sky blue) performed at 13:00 of diestrus 1 (D1), diestrus 2 (D2) or proestrus (P) sacrificed one hour after surgery. * p < 0.05 vs. unilateral perforation of the peritoneum (Student's *t *test).

Figure 2 shows that the effects of blocking the cholinergic system of rats with unilateral perforation of the peritoneum depended on both, the side (left or right) and the phase of the estrous when surgery was performed. Injecting ATR on D1 or D2 resulted in a significant drop in E_2 _serum concentrations in animals with sham treatment on the right side. Blocking the cholinergic system of rats with left side peritoneum perforation on D1 or P resulted in a drop in E_2 _serum concentrations (52%; 47%, respectively), while the same treatment performed on D2 resulted in a significant E_2 _concentrations increase (157%).

**Figure 3:**
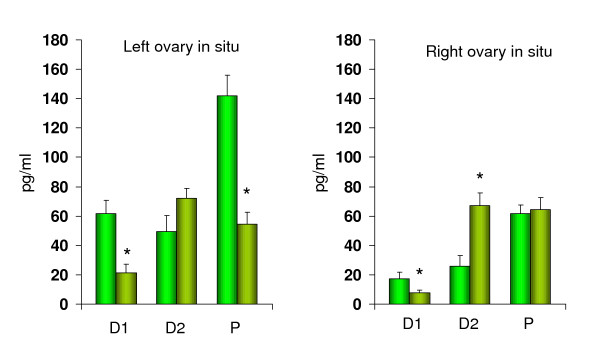
Comparative effects of atropine sulfate injection at 12:00 hours on estradiol serum levels (pg/ml) to rats with unilateral ovariectomy (olive green) performed at 13:00 of diestrus 1 (D1), diestrus 2 (D2) or proestrus (P) sacrificed one hour after surgery. * p < 0.05 vs. unilateral ovariectomy (Student's *t *test).

Figure 3 shows the effects of blocking the cholinergic system of rats with ULO. ATR treatment on D1 or P stages performed on rats with the left ovary *in situ *resulted in a significant drop of E_2 _serum concentrations (65%; 62% respectively). Such effects were not observed in rats treated on D2. When ATR treatment was performed on rats with the right ovary *in situ *on D1, E_2 _serum concentrations were lower (48%) than in ULO animals. Blocking the cholinergic system on D2 resulted in E_2 _serum concentrations increase (159%). When the treatment was performed on P, no significant differences in E_2 _serum concentrations were observed.

Compared to bilateral treatment, perforation of the peritoneum or bilateral ovariectomy, ATR treatment on D1 resulted in significant E_2 _serum concentrations decreases, 90% in bilateral peritoneal perforation and 60% in bilateral ovariectomized animals.

**Figure 4:**
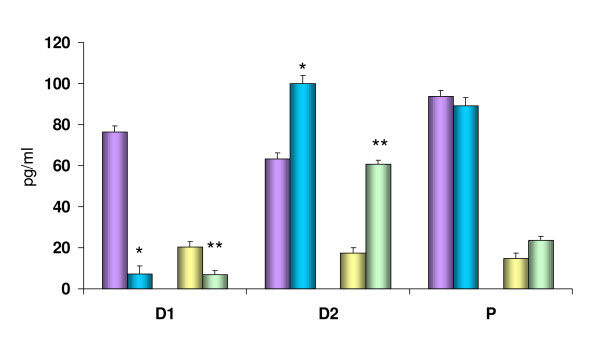
Comparative effects of atropine sulfate (blue, green) injection at 12:00 hours on estradiol serum levels (pg/ml) to rats with bilateral perforation of the peritoneum (purple) or bilateral ovariectomy (yellow) performed at 13:00 of diestrus 1 (D1), diestrus 2 (D2) or proestrus (P) sacrificed one hour after surgery. * p < 0.05 vs. bilateral perforation of the peritoneum (Student's *t *test); ** p < 0.05 vs. bilateral ovariectomy (Student's *t *test).

Blocking the cholinergic system on D2, to rats with bilateral perforation of the peritoneum or bilateral ovariectomy resulted in E_2 _serum concentrations increases (159% and 253% respectively), while injecting ATR to animals treated on P had no apparent effects (Figure 4).

## Discussion

The results obtained in the present study suggest that the ability to compensate the secretion of E_2 _by the missing ovary is different between the right and left ovaries and varies during the estrous cycle. Similarly, our results suggest that the cholinergic system participates in regulating E_2 _secretion by the ovary, and that such participation varies depending on the ovary remaining *in situ *and the stage of the estrous cycle when the surgical procedure was performed.

Previously, we suggested the existence of a neural pathway arising from the peritoneum that participates in regulating E_2 _[9], P_4 _[10] and testosterone secretion [11]. In the rat, the sensory information arising from the peritoneum is sent to the nucleus tractus solitarius and stimulates neurokinine-B receptors [17]. Since perforating the peritoneum unilaterally on each day of the estrous cycle changed E_2 _serum concentrations, we think that each side of the peritoneum sends different neural information through the superior ovarian nerve (SON) to the ovary and the central nervous system, perhaps reaching nuclei related to the vagus nerve.

A study analyzing the distribution of sensory neurons innervating the peritoneum showed that when tracer was placed on the area where the peritoneum covers the abdominal wall, labeled neurons were observed only in the ipsilateral dorsal root ganglia [18]. The authors suggest that most of the parietal peritoneum receives sensory nerves from dorsal root ganglia, and visceral peritoneum from both, the spinal and vagus nerves.

According to Stener-Victorin et al. (19) repeated electro-acupunture treatments in rats with polycystic ovary syndrome (PCO), induced by a single injection of estradiol valerate, resulted in lower nerve growth factor (NGF) concentrations at the ovarian level than in non-electro-acupunture treated PCO rats. In our experiments, perforating the peritoneum affected the same somatic segments employed by Stener-Victorin et. al. [19].

We presume that peritoneum surgery resulted in an increase of NGF concentrations at the ovarian level, which in turn induced hyper-androgenism, as observed in women with PCO [20]. Previously, we showed that the unilateral perforation of the peritoneum results in a significant increase in testosterone serum concentrations [11]. Because E_2 _serum concentrations did not increase after left or bilateral perforation of the peritoneum, we suppose that the neural information originating from the peritoneum inhibits the mechanisms regulating aromatase activity within the follicle.

One of the ovaries' sources of catecholamines arrives through the SON. In the ovary, the SON fibers are mainly distributed in the peri-follicular theca layer, and in close relation with the cells of the theca interna [21,22]. Sectioning the SON of rats in P results in a sudden drop of P_4 _and E_2 _concentrations in the ovarian vein effluent [23], while the same procedure on estrus did not modify E_2 _concentrations [24]. Therefore, it is possible that perforating the peritoneum modifies the type and/or rate of information arriving to the ovary via the SON. Another possibility is that perforating the left side of the peritoneum results in an increase release of ovarian gamma amino butyric acid (GABA), and a subsequent increase of E_2 _concentrations.

According to Erdö, et. al. [25] and Laszlo, et. al. [26], injecting GABA into pseudo-pregnant rats increases E_2 _concentration in the blood. Present results indicate that injecting ATR, before unilateral or bilateral perforation of the peritoneum, to rats in D1 or P, results in lower E_2_serum concentrations; leading us to think that some of the neural fibers present in the peritoneum are muscarinic. It is also possible that blocking the cholinergic innervation, by ATR treatment, results in lower adrenaline and norepinephrine release by the adrenal medulla.

Our results, and those of others, suggest that stimulating on D2 the sensory receptors located on the left side of the peritoneum triggers an E_2 _secretion inhibitory mechanism, that the sensory pathway arising from the right side has a stimulatory effect, and that both are mediated by the cholinergic muscarinic system.

Previously, we showed that injecting ATR to rats in D2 results in increases of P_4 _serum concentrations originating from the adrenals [8], without having apparent effects on testosterone serum concentrations [11]. Since P_4 _and androgens are precursors in the synthesis of E_2_, we presume that this mechanism may explain the increase in E_2 _serum concentrations observed in rats with peritoneum perforation previously injected with ATR.

Another possibility explaining the differences on E_2 _secretion regulation during the estrous cycle is that the effects of the cholinergic system take place through changes at the celiac ganglion level. According to Aguado and Ojeda [23], acetylcholine inhibits P_4 _secretion in the celiac ganglion-SON-ovary preparation obtained from rats in D1 or D2, while the preparation obtained from rats in P resulted in only a moderate stimulation. Since there is evidence that fibers from the vagus nerve innervate neurons in the celiac ganglion [27], we presume that the cholinergic system modulates the sympathetic post-ganglionar activity and the secretory ability of the ovaries through the SON.

## Conclusion

Based on the differences in E_2 _serum concentrations in rats with ULO, present results suggest that the capacity to release E_2 _by the left and right ovaries varies during the estrous cycle. We presume that the left ovary releases more E_2 _than the right one. As previously proposed, another possibility is that neural communication between the ovaries modulates E_2 _secretion.
